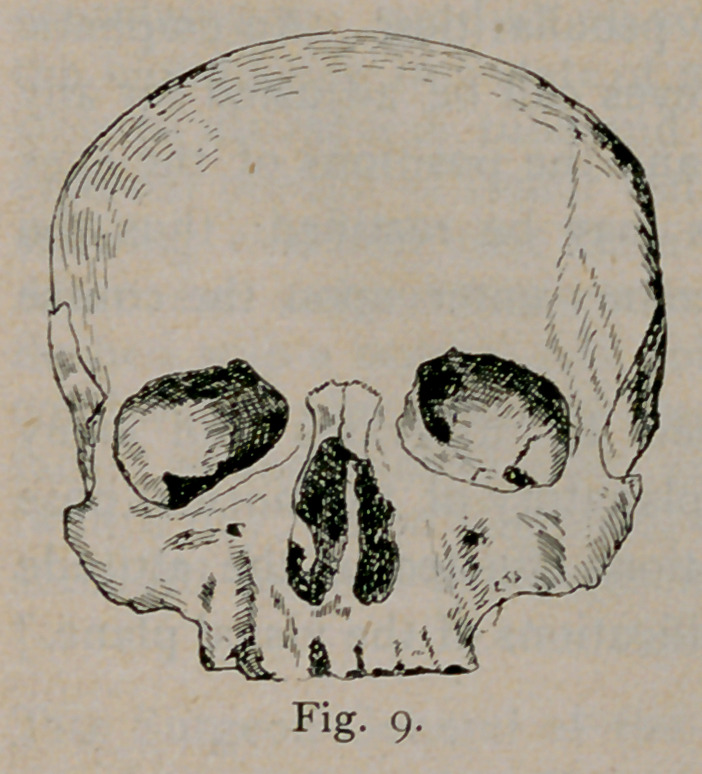# The Pose of the Body as Related to the Type of the Cranium and the Direction of the Visual Plane1Address delivered before the Section of Ophthalmology, Otology and Laryngology, Buffalo Academy of Medicine, January 21, 1901.

**Published:** 1901-03

**Authors:** George T. Stevens

**Affiliations:** New York


### THE POSE OF THE BODY AS RELATED TO THE TYPE OF THE CRANIUM AND THE DIRECTION OF THE VISUAL PLANE.[1]

By GEORGE T. STEVENS, M. D., New York.

[Author's Abstract.]

IT IS a novel proposition that the position of the head in respect to the body or of the shoulders in reference to the back, that the carriage of the whole body in walking and the attitude of one in conversation, should be governed in an important measure by the form of the cranium. It will also, doubtless, be regarded as a bold assertion to say that all these positions and attitudes and even the gait of the individuals are largely modified, even in many instances controlled, by the normal position of the eyes in respect to the cranium.

1. Address delivered before the Section of Ophthalmology, Otology and Laryngology, Buffalo Academy of Medicine, January 21, 1901.

Yet it is not difficult to show that both these propositions are true and that the truth contained in them is not only of importance as a principle in art, but that it is of great practical value from the point of view of the well being of large groups of persons.

From the practical standpoint it may be said that, owing to the position of the visual plane in respect to the head, there may be comparative immunity from certain complaints and diseases, or a comparative predisposition to these very affections.

By the term "normal plane of vision" it is intended to express the direction of the lines of sight, the eyes being in a passive condition and the head being in "the primary position."

Under these conditions the lines of sight lie in an imaginary

Fig. 1.

Fig. 2.

Fig. 3.

plane which may be coincident with, or somewhat higher than, or lower than, the plane of the horizon.

The form of the head of an individual is not always so clearly of one or another type that it must be classed as within a precise group, yet, in general, it may be said that heads are grouped by craniologists in three great classes or types. There are also sub-types, which need not now be considered. The types of crania are, the long, the broad and the medium, or, for this discussion, the tall.

Long heads have the transverse diameter, 75-100 that of the length, or less than that. In broad skulls the transverse diameter is 85-100 that of the longest diameter, or more, while medium skulls have an index between these. The series of diagrams, Figs. 1, 2 and 3, give the general outline of these types as seen from above.

In the next series of diagrams, Figs. 4, 5 and 6, is presented the idea of the general form of the head in each of these classes from a side view in living subjects. The normal visual plane does not occupy the same position in relation to the horizon in these three classes of heads.

Fig. 4.

Fig. 5.

This can be determined in the living subject by the aid of the tropometer. In the prepared skull it can be determined by finding the direction of the orbital axis by a method which I have described.

Fig. 6.

The form of the orbit in the different classes of skulls offers an explanation of the peculiarities in the direction of the orbital axis, as well as of the normal plane of vision.

Figs. 7, 8 and 9 represent front views of skulls of the long, tall and broad types, respectively. In the long skull the roof of the orbit is low and the floor is low, the axis points down. In the tall skull the roof of the orbit is high and the axis of the orbit is materially higher than in the other type. In the broad skulls the roof, as well as the floor is low and the direction of the transverse diameter of the orbital opening is oblique. The orbital axis points down.

Associated with the cranial index, the proportion between the long and short diameters of the skull, there is an important modifying feature in the facial angle. If, with the long skull, the facial angle is external (Fig. 4) and of a pretty high degree, the plane of

Fig. 7.

Fig. 8.

vision is low. In case of the tall skull with slight facial angle (Fig. 5) the plane is high. In the case of the broad skull with inverse facial angle (Fig. 6) the plane is low.

The person whose normal plane of vision is quite low throws the head back, lifting the chin (Figs. 4 and 6). On the contrary, the person whose plane of vision is normally high, throws the forehead in advance and the chin into the breast. (Fig. 5.) It is easy to see that this selection of an easy method of adjusting the lines of sight to surrounding objects, exercises a commanding influence on the whole pose of the body. Other elements which enter into the case may give rise to exceptions. These elements are, when they occur, quite evident and can be found. The exceptions can therefore be explained.

Fig. 9.

When the visual plane is quite low not only is the chin elevated but the muscles of the whole back, even those of the lower part, are habitually put in a state of tension. Hence, among the delicate people who have this peculiarity it is common to find pains in the muscles of the back of the neck, in the small of the back, and even in the lumbar region. In these people the brows are strongly elevated and there are other characteristic facial expressions. People with this class of head are more liable than others to certain physical complaints and nervous disturbances. They are also, to a large extent, immune to certain other forms of affections. Negroes of Guinea coast ancestry are less subject to consumption than the whites in similar localities in the United States. When they have not too great a mixture of white blood they have long heads, with very marked external facial angle. They have therefore a low visual plane and carry the chin high in the air.

Among the inhabitants of Iceland, who have very broad heads, and low visual plane, and who carry the chin very high, consumption is unknown, although their mode of living is most unsanitary.

People with tall heads and with faces with a very low angle or no angle have the brows strongly depressed and carry the forehead in advance (Fig. 5.) Their shoulders often bend forward, the chest is compressed, and their walk is characteristic. In this class is found the great majority of cases of consumption.

Some years ago the speaker called attention to the one-sided carriage of the head in those in whom one visual line tends higher than the other. In such cases, especially in those in which are also found certain forms of "declination," torticolis or scoliosis may and often does result.

The practical question which arises from the presentation of this subject is: can the direction of the visual plane be so modified as to change the pose of the body to advantage and thereby relieve the victim from the results of their physical peculiarities? An emphatic affirmative answer can be made. The eyes can be adjusted for any plane by a safe and speedy procedure, and the positions of the eyes due to the hereditary form of the orbits may be rectified: thus the handicap with which large classes of persons enter upon the course of life may be removed.

[NOTE.—The address was illustrated by diagrams and many photographs thrown upon the screen explanatory of the normal pose in the various classes, and the modifications effected in the attitude and carriage as the result of proper modifications of the visual plane.]

---

THE sanatorium for consumptives, which was established by the government at Fort Stanton, New Mexico, has been a great success. In the first year and a half of its existence ninety-two patients were admitted from various marine hospitals, twelve of whom were discharged as recovered and fifteen as improved.—*Medical Age.*

## Figures and Tables

**Fig. I. f1:**
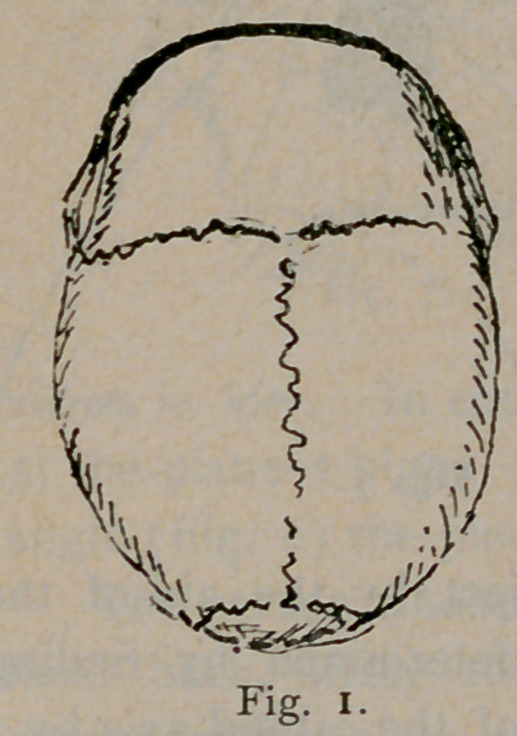


**Fig. 2. f2:**
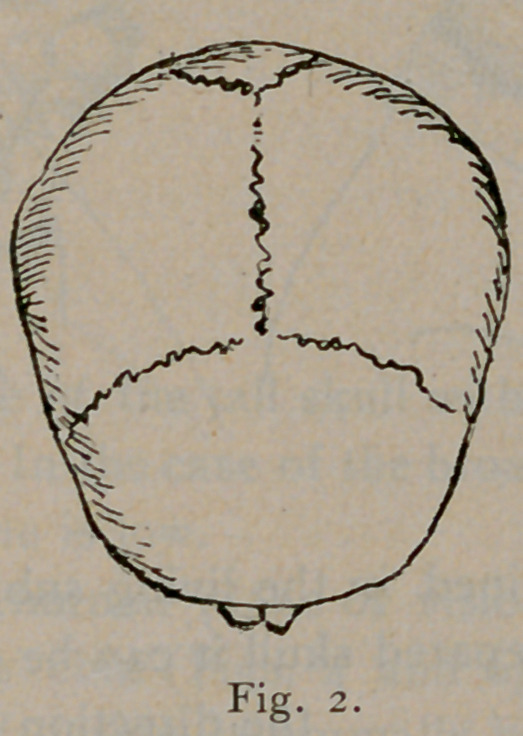


**Fig. 3. f3:**
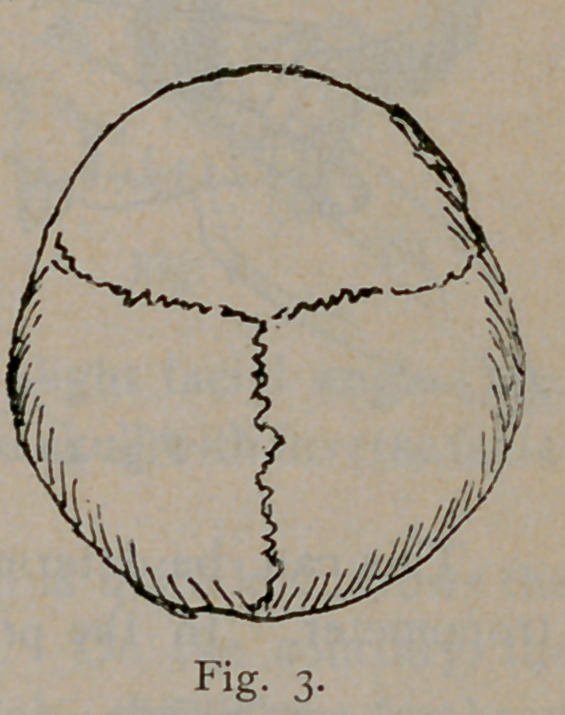


**Fig. 4. f4:**
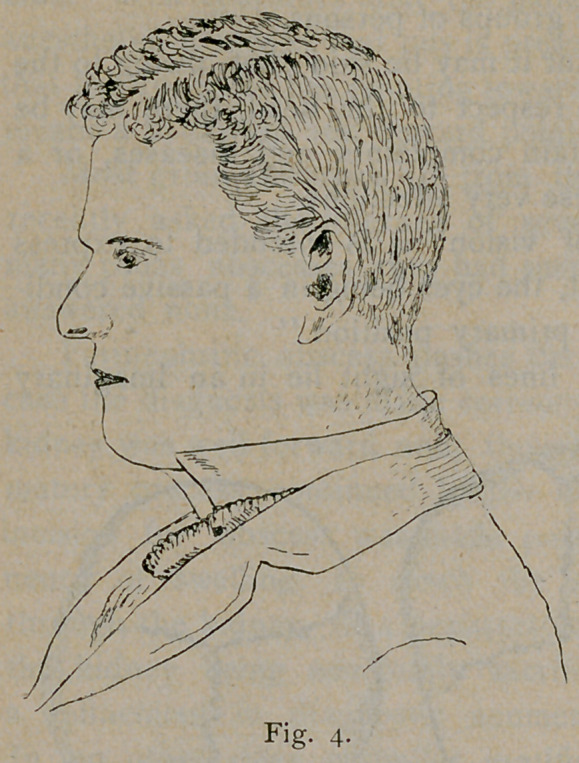


**Fig. 5. f5:**
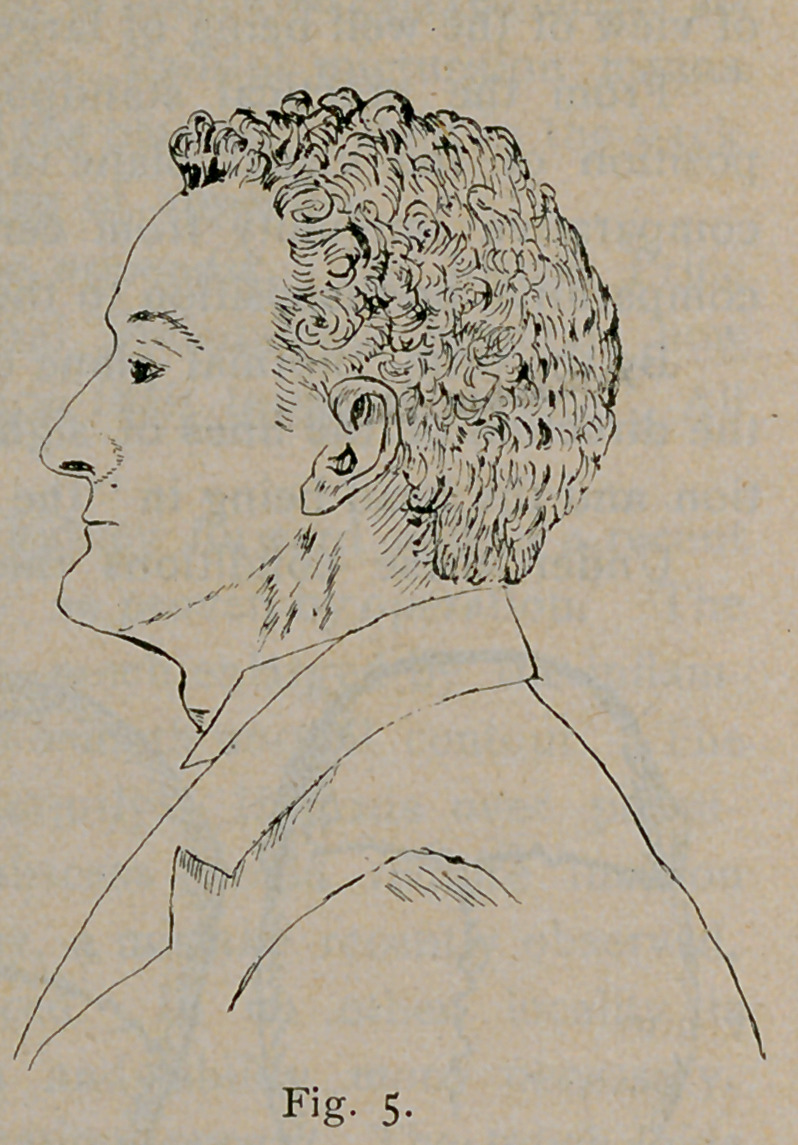


**Fig. 6. f6:**
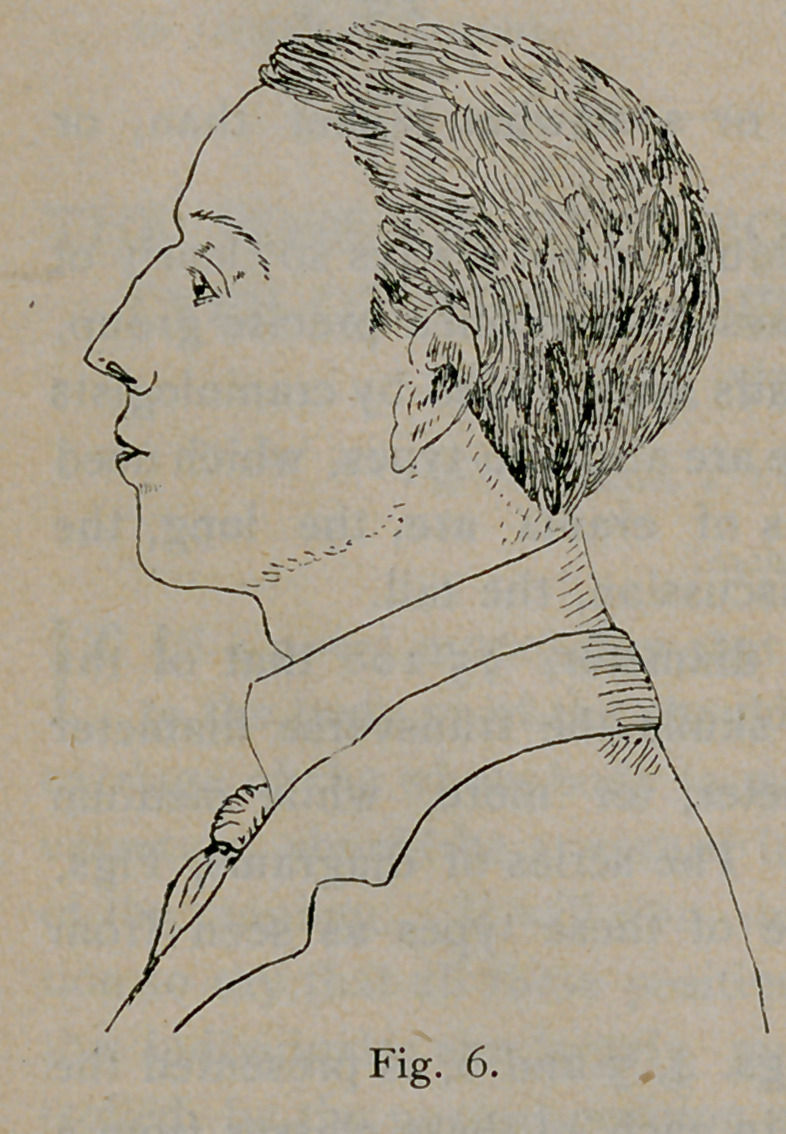


**Fig. 7. f7:**
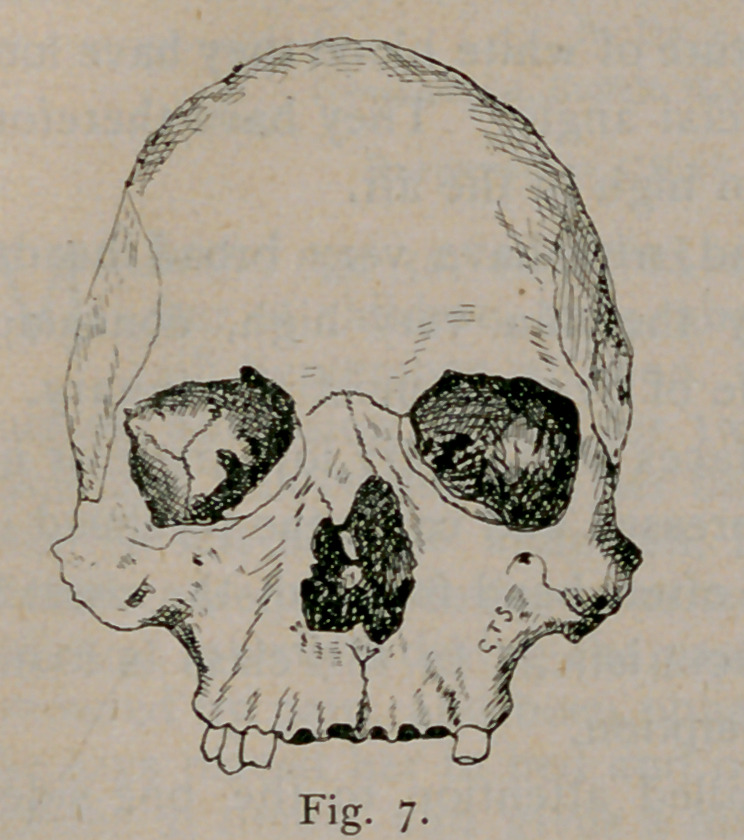


**Fig. 8. f8:**
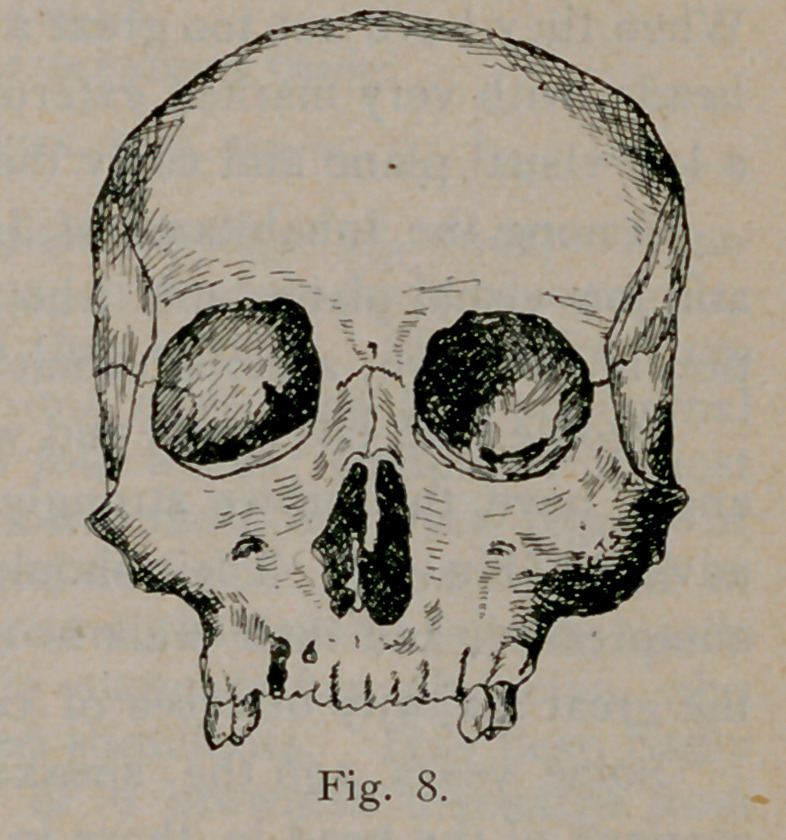


**Fig. 9. f9:**